# The stabilizing effects of genetic diversity on predator-prey dynamics

**DOI:** 10.12688/f1000research.2-43.v1

**Published:** 2013-02-12

**Authors:** Christopher F Steiner, Jordan Masse

**Affiliations:** 1 Department of Biological Sciences, Wayne State University, Detroit, MI, 48202, USA

## Abstract

Heterogeneity among prey in their susceptibility to predation is a potentially important stabilizer of predator-prey interactions, reducing the magnitude of population oscillations and enhancing total prey population abundance. When microevolutionary responses of prey populations occur at time scales comparable to population dynamics, adaptive responses in prey defense can, in theory, stabilize predator-prey dynamics and reduce top-down effects on prey abundance. While experiments have tested these predictions, less explored are the consequences of the evolution of prey phenotypes that can persist in both vulnerable and invulnerable classes. We tested this experimentally using a laboratory aquatic system composed of the rotifer *Brachionus calyciflorus* as a predator and the prey *Synura petersenii*, a colony-forming alga that exhibits genetic variation in its propensity to form colonies and colony size (larger colonies are a defense against predators). Prey populations of either low initial genetic diversity and low adaptive capacity or high initial genetic diversity and high adaptive capacity were crossed with predator presence and absence. Dynamics measured over the last 127 days of the 167-day experiment revealed no effects of initial prey genetic diversity on the average abundance or temporal variability of predator populations. However, genetic diversity and predator presence/absence interactively affected prey population abundance and stability; diversity of prey had no effects in the absence of predators but stabilized dynamics and increased total prey abundance in the presence of predators. The size structure of the genetically diverse prey populations diverged from single strain populations in the presence of predators, showing increases in colony size and in the relative abundance of cells found in colonies. Our work sheds light on the adaptive value of colony formation and supports the general view that genetic diversity and intraspecific trait variation of prey can play a vital role in the short-term dynamics and stability of planktonic predator-prey systems.

## Introduction

A large body of theoretical and empirical work has shown that the presence of variation among prey in their susceptibility to predation can have profound impacts on the structure and dynamics of predator-prey communities ^1– 9^. Prey heterogeneity can mediate trophic-level responses to enrichment and weaken top-down limitation of prey communities by facilitating numerical dominance by defended prey ^2, 4, 7, 10– 12^. Such shifts in dominance can have significant dynamic consequences, potentially reducing the propensity and severity of predator-prey oscillations and stabilizing community dynamics ^1, 3, 5, 9^. While empirical research has largely focused on the consequences of interspecific variation in prey edibility, a growing body of experiments has begun to highlight the dynamic consequences of intraspecific trait variation. When prey populations exhibit phenotypic variation in their susceptibility to predators and show rapid adaptive responses to predation pressure, they can fundamentally alter the strength and dynamic consequences of their interactions with their consumers ^13– 18^.

The effects of prey heterogeneity on predator-prey dynamics has been extensively explored in the context of endogenously driven population cycles. Cycles are a common feature of simple, two-species predator-prey models that incorporate nonlinear functional responses ^19– 22^. Such oscillatory dynamics become more probable and increase in amplitude with increasing prey carrying capacity ^20, 21^. Inclusion of prey heterogeneity in the form of species that are defended from predation can in theory stabilize predator-prey cycles. This readily occurs when prey species trade off their ability to compete for shared resources with their capacity to resist predation. In such instances, predators can facilitate the invasion and persistence of defended prey which, in turn, siphon resources from more edible species, reducing their carrying capacity. This can, in some cases, decrease the amplitude of predator-edible prey cycles or move systems from periodic to point attractors ^3, 5, 23^. Predator-mediated increases in the relative abundance of defended prey may also weaken top-down limitation of trophic-level abundance causing an increase in total prey abundance relative to prey community’s lacking trait heterogeneity ^2, 4, 7, 10– 12^.

While the abovementioned models were developed with the intent of understanding the dynamic consequences of heterogeneity among prey species, their general predictions may, under certain conditions, apply to heterogeneity that occurs within species. For instance, models of trophic structure that incorporate prey evolutionary dynamics show that heritable variation in defense against predators may allow prey populations to adaptively respond to predation pressure, shifting regulation of prey populations from top-down control to stronger bottom-up control ^13, 24^. Several models have also explored how adaptive responses in prey defense may impact predator-prey dynamics and stability ^14– 16, 18, 24– 26^. These demonstrate the capacity for prey evolution to dramatically alter predator-prey dynamics but stabilization is not a generalizable prediction. While adaptive responses in prey defense may stabilize predator or prey populations under certain conditions ^14, 15, 24^, evolution can also give rise to destabilization of predator-prey cycles and enhanced extinction probability ^14, 16, 17, 25, 26^.

Only a handful of studies have attempted to address the dynamic consequences of intraspecific variability in prey edibility using direct manipulations in which prey evolution was either suppressed xor promoted ^16, 17, 27^. Moreover, previous experiments have only considered phenotypes or species with fixed traits. For many organisms, ecological strategies may involve transitions between dynamic classes that vary in their susceptibility to predators, with variation among phenotypes consisting of variation in state transition rates or degree of invulnerability. This could apply to organisms that change behavioral/physiological states or who move in and out of spatial refuges. It could also occur with colony-forming organisms in which colonies of increasing size are more resistant to predators. Prior work has shown that such prey strategies can stabilize predator-prey cycles ^1^. However, this has not been examined within an evolutionary context.

**Figure 1.**
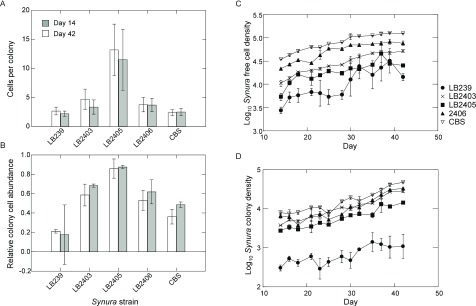
Results of short-term assays examining trait variation among the five *Synura* strains used in the experiment. Source cultures were maintained under common garden conditions using the same environmental conditions as the main experiment. Individuals of each *Synura* strain were isolated from one week old cultures and used to establish three replicate monocultures at an initial total cell density of 1500 cells/mL. Monocultures experienced the same environmental conditions (light, temperature and nutrient replacement) and sampling procedures as the main experiment. ( **A**) Mean cells per colony of each strain estimated from samples taken on day 14 and day 42 of the assay (shown are means and 95% confidence intervals). ( **B**) Mean relative abundance of cells found in colonies estimated from samples taken on day 14 and day 42 of the assay (shown are means and 95% confidence intervals). ( **C**) Density of free-living cells over time for each strain. Shown are means (+/- S.E.); original units in numbers of individuals per mL. ( **D**) Density of colonies over time for each strain. Shown are means (+/- S.E.); original units in numbers of colonies per mL.

We tested this experimentally using a laboratory-based aquatic system composed of the zooplankton-predator *Brachionus calyciflorus* and the algal-prey *Synura petersenii*, in which the potential for prey evolution was either enhanced or reduced through direct manipulations of initial prey genetic diversity. Our study differed from prior work in its use of an algal-prey species that can transition between a vulnerable and predator-resistant class. *Synura petersenii* is a common freshwater flagellate that may transition between two states: either free-living cells (which are more susceptible to zooplankton predators) or as swimming colonies (which are less susceptible to zooplankton feeding due to their larger size). Reproduction can occur in either the free-living state or in the colony state through binary fission. The strains of *S. petersenii* used in our study exhibit heritable variation in their propensity to form colonies and their degree of vulnerability when in the colony state due to variation in colony size ( Figure 1). Cellular aggregation and colony formation are viewed as key steps in the evolution of multicellularity ^28– 30^. Thus, our work also permits exploration of the selective forces that may favor colonial strategies. We show that the initial presence of trait heterogeneity among prey can reduce top-down limitation of prey and alter predator-prey dynamics by reducing temporal variation in total prey abundance. This stabilization is associated with an increase in the size of colonies and the relative abundance of cells found in colonies.

## Methods

Our experimental system consisted of a single species of zooplankton as a predator, the rotifer *Brachionus calyciflorus*, and a single species of phytoplankton as the prey, the colony-forming flagellate *Synura petersenii*. Hereafter we refer to both species by genus. *Brachionus* cultures were obtained from Florida Aqua Farms (Dade City, FL, USA). Five strains of *Synura* were used in the experiment, four of which (LB239, LB2403, LB2405, LB2406) were obtained from UTEX (Austin, TX, USA) and one (CBS) from Carolina Biological Supply (Burlington, NC, USA). *Synura* stock cultures were initiated with a single cell isolated from serial dilutions in sterile medium to ensure that all stocks were initially isogenic. All five *Synura* strains produced populations composed of a mix of single cells and colonies when under semi-continuous culture conditions. However, short-term trait assays revealed significant genetic variation among the strains in population densities, colony size (number of cells per colony), and the relative abundance of total cells found in colony form ( Figure 1). *Brachionus* used in the experiment were isolated from a clonal culture grown on the CBS strain of *Synura*. This culture was initially stocked with a single *Brachionus* clone, minimizing genetic variation within our *Brachionus* populations and reducing the potential for coevolution between predator and prey. All stock cultures were maintained using the same environmental conditions as in the experiment.

All experimental materials were autoclave-sterilized prior to use. Experimental containers consisted of 500mL flasks filled with 400mL of COMBO medium ^31^, capped with aluminum foil and housed in a single environmental chamber at 20°C under 24 hour light. The containers were randomly ordered and rotated in the chamber following each sampling event. We used a factorial design in which predator presence/absence was crossed with a manipulation of initial genetic diversity (a low genetic diversity treatment composed of each *Synura* strain in monoculture or a high genetic diversity treatment composed of all five strains together). The treatments with *Brachionus* present were replicated four times; treatments without *Brachionus* were replicated three times. At the initiation of the experiment, flasks were first inoculated with their respective *Synura* strains from stock cultures. The high genetic diversity treatment received 20 cells/mL of each of the five strains while the low diversity treatment received a single strain at 100 cells/mL. Thus, total *Synura* density added was kept constant at 100 cells/mL across treatments. While the five strains were added at lower initial densities in the high genetic diversity treatment, each flask received a total of 8000 cells of each strain. Hence, the probability of losing a strain through demographic stochasticity and genetic drift at the initiation of the experiment was low. *Synura* populations were allowed to grow in the absence of predators for seven days at which time 10 *Brachionus* adults were added to each plus predator treatment. We refer to this as day 0 of the experiment. *Brachionus* populations were allowed to grow for 12 days (with periodic medium replacement) at which time sampling was initiated.

Sampling occurred every 2–3 days up to day 167, the final day of the experiment. To sample the experiment, flasks were first gently swirled to homogenize their contents and 12.5% of the volume (50mL) was poured into a sample bottle, which served as both a zooplankton and phytoplankton sample. Removed medium was replaced with sterile COMBO medium. Thus, the experiment was maintained under semi-continuous culture conditions. *Brachionus* was enumerated using a stereomicroscope while *Synura* was enumerated using a CASY particle counter (Innovatis AG, Germany). Maximum cell diameter of *Synura* in our cultures ranged between 6–10µm (mean=7.9µm); we found no significant differences among our strains in mean maximum diameter. Consequently, we programmed the CASY to perform total counts and counts of particles below 10.5µm mean diameter as an estimate of the density of free living cells. Colony densities were then calculated by subtracting single cell counts from totals. To measure the density of cells in colonies, we multiplied the colony counts by estimates of the mean number of cells per colony for each treatment replicate. Estimates of cells per colony were performed at four time points during the experiment: at the initiation of the experiment, on day 30, day 97 and at the end of the experiment. To obtain estimates, subsamples of phytoplankton from each replicate were preserved in acid Lugols solution and counts performed using a compound microscope. We counted cells/colony for up to 25 haphazardly chosen colonies per sample and averaged the values. Because we did not have mean colony size estimates for all dates, we time-averaged the estimates of cells/colony over days 30 to 167 to create conversion constants for each replicate prior to multiplying by the colony counts. This should have provided conservative estimates of treatments effects since treatments had not completely diverged by the day 30 sample. Total cell density was calculated by summing free-living cell densities with estimates of the density of cells found in colonies.

*Brachionus* populations in the low diversity treatment rapidly went extinct in all replicates of the LB239, LB2403, LB2405, and LB2406 strains. We consequently discontinued sampling of these monocultures and focused on the CBS strain for the low genetic diversity treatment. *Brachionus* populations exhibited an initial exponential growth phase followed by a population decline between days 0 and 40 before settling into more regular population oscillations. To remove the influence of initial transitory dynamics we analyzed data over days 51 to 167. Significant linear trends over time in *Synura* and *Brachionus* densities were evident in several replicates (see Results). To remove the influence of temporal trends on measures of temporal variability, we first performed linear regressions of log transformed densities versus time for each replicate for both species. Mean absolute values of the regression residuals were then used as measures of temporal variability (an inverse measure of stability). Analyses of mean *Brachionus* and *Synura* densities were performed on log _10_ transformed values averaged over days 51 to 167. To analyze changes in the degree of colony formation, we examined colony densities and the relative abundance of cells found in colonies (equal to the density of cells in colonies divided by total cell density). Response variables were analyzed using ANOVA. Because replication was not equal among replicates, we used type III sums of squares and max t-tests for post hoc comparisons, which are robust to unbalanced designs and non-normal data ^32^. All response variables met assumptions of normality using Lilliefor’s test. Statistics were performed in R version 2.15 ^33^. Code for running max t-tests can be found in ^32^.

## Results

**Figure 2.**
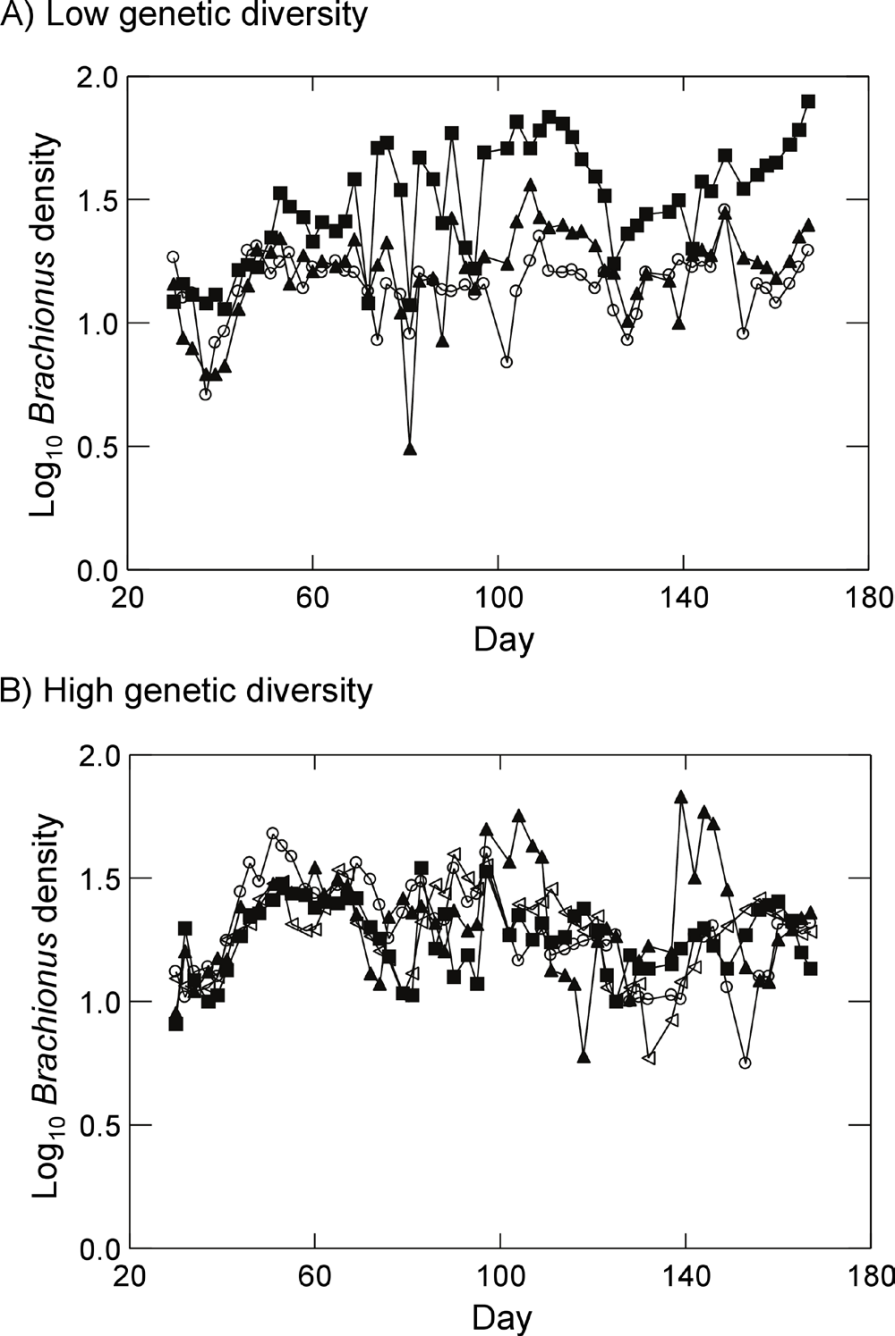
*Brachionus* dynamics in the presence of ( **A**) low initial prey genetic diversity (prey populations started with a single strain of *Synura*) or ( **B**) high initial prey genetic diversity (prey population started with five strains of *Synura*). Dynamics for each replicate are shown separately (different symbols). Original units in numbers of individuals per mL.

**Figure 3.**
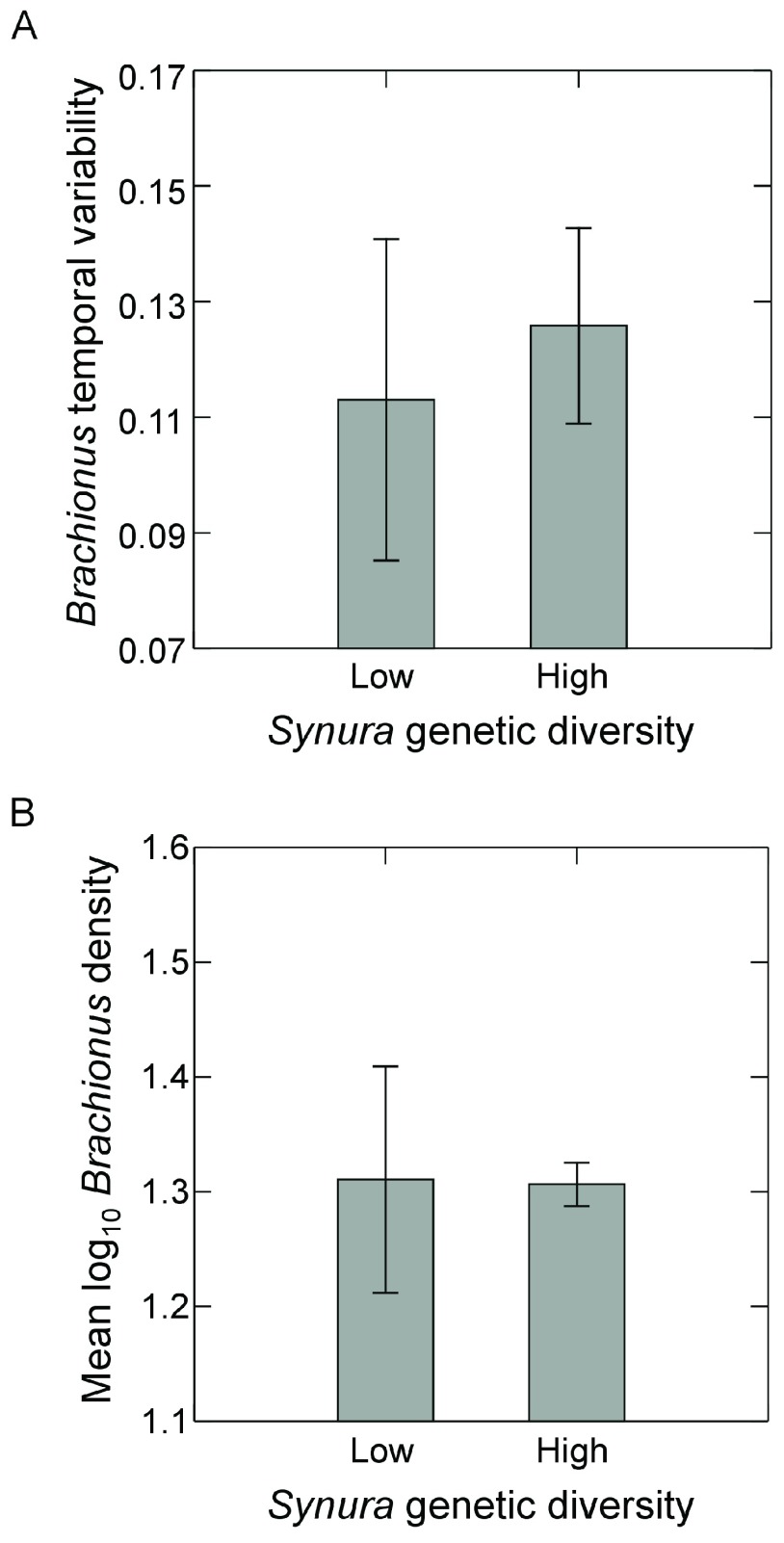
Effects of initial *Synura* genetic diversity (low versus high) on ( **A**) detrended temporal variability of *Brachionus* populations (an inverse measure of stability), measured as mean residuals from linear regressions of log transformed *Brachionus* density versus time, and ( **B**) *Brachionus* densities averaged over the course of the experiment. Both measures ( **A** and **B**) were based on dynamics over days 51 to 167. Shown are means (+/- S.E.). Original units in numbers of individuals per mL.

*Brachionus* populations persisted in all high genetic diversity treatments. However, populations of the zooplankton failed to establish in all low diversity replicates that were composed of the LB239, LB2403, LB2405, and LB2406 genotypes (sampling of these monocultures was discontinued). *Brachionus* also went extinct mid-experiment in one low genetic diversity replicate containing the CBS strain; we have removed this replicate from all analyses. Figure 2 displays *Brachionus* dynamics for all replicates in the presence of either low initial genetic diversity ( Figure 2A) or high initial genetic diversity ( Figure 2B). When examining dynamics from day 51 to day 167, linear regressions revealed a significant positive trend in log *Brachionus* densities over time in one low genetic diversity replicate (p=0.02). Three of four high genetic diversity replicates exhibited significant negative trends in log *Brachionus* densities over time (p<0.05, linear regression). Examining regression residuals, *Synura* genetic diversity had no effect on detrended temporal variability of *Brachionus* ( Figure 3A; p=0.62, ANOVA). Time-averaged *Brachionus* densities also showed no responses to initial *Synura* genetic diversity ( Figure 3B; p=0.93, ANOVA).

**Figure 4.**
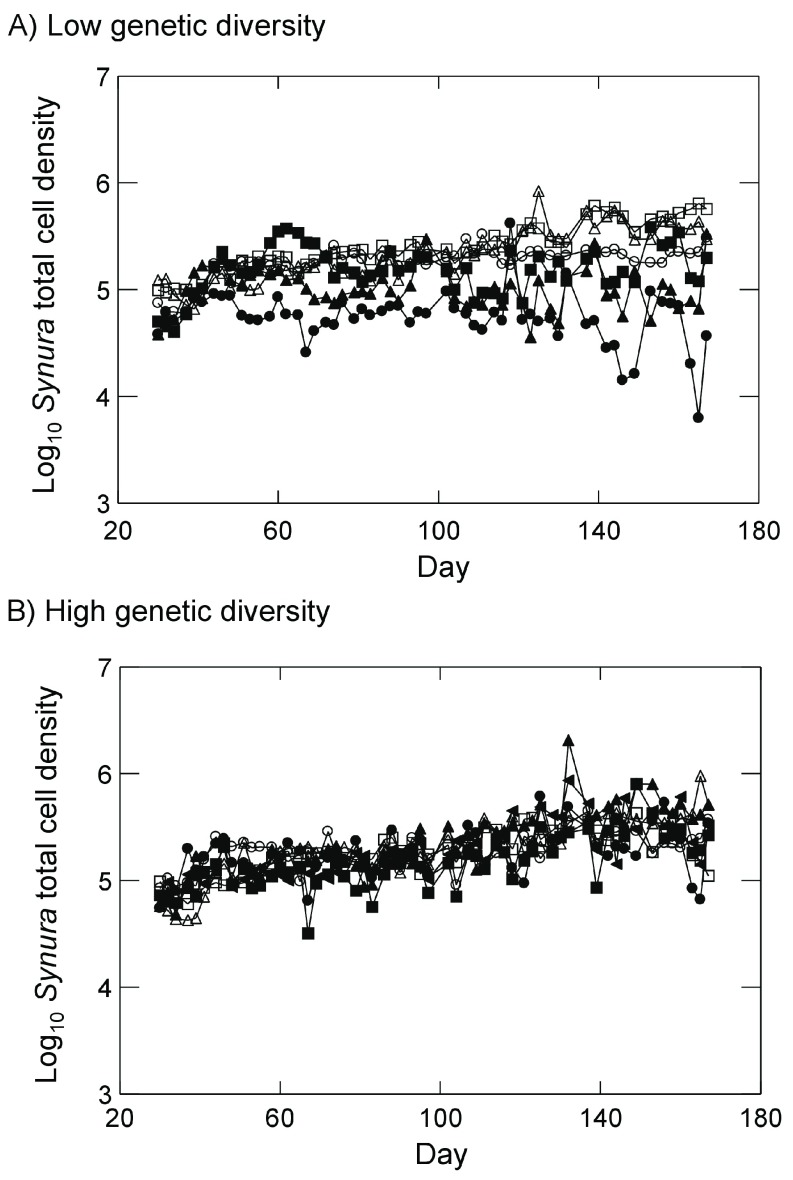
*Synura* dynamics in presence of *Brachionus* (closed symbols) or absence of *Brachionus* (open symbols) for ( **A**) populations with low initial *Synura* genetic diversity or ( **B**) populations with high initial *Synura* genetic diversity. Dynamics for each replicate are shown separately (different symbols). Original units in numbers of individuals per mL.

*Synura* dynamics for all treatments and replicates are shown in Figure 4. Examining dynamics from day 51 to day 167, log *Synura* densities in the low genetic diversity treatment and in the presence of *Brachionus* showed no significant trends with time across all three replicates ( Figure 4A; all p<0.08, linear regressions). By contrast, in the absence of *Brachionus*, *Synura* densities in the low genetic diversity treatments showed significant positive trends with time in all replicates ( Figure 4A; all p<0.001, linear regressions). Turning to *Synura* dynamics in the high genetic diversity treatments, all replicates exhibited significant positive increases over time in both the presence and absence of *Brachionus* ( Figure 4B; all p<0.019, linear regressions).

**Figure 5.**
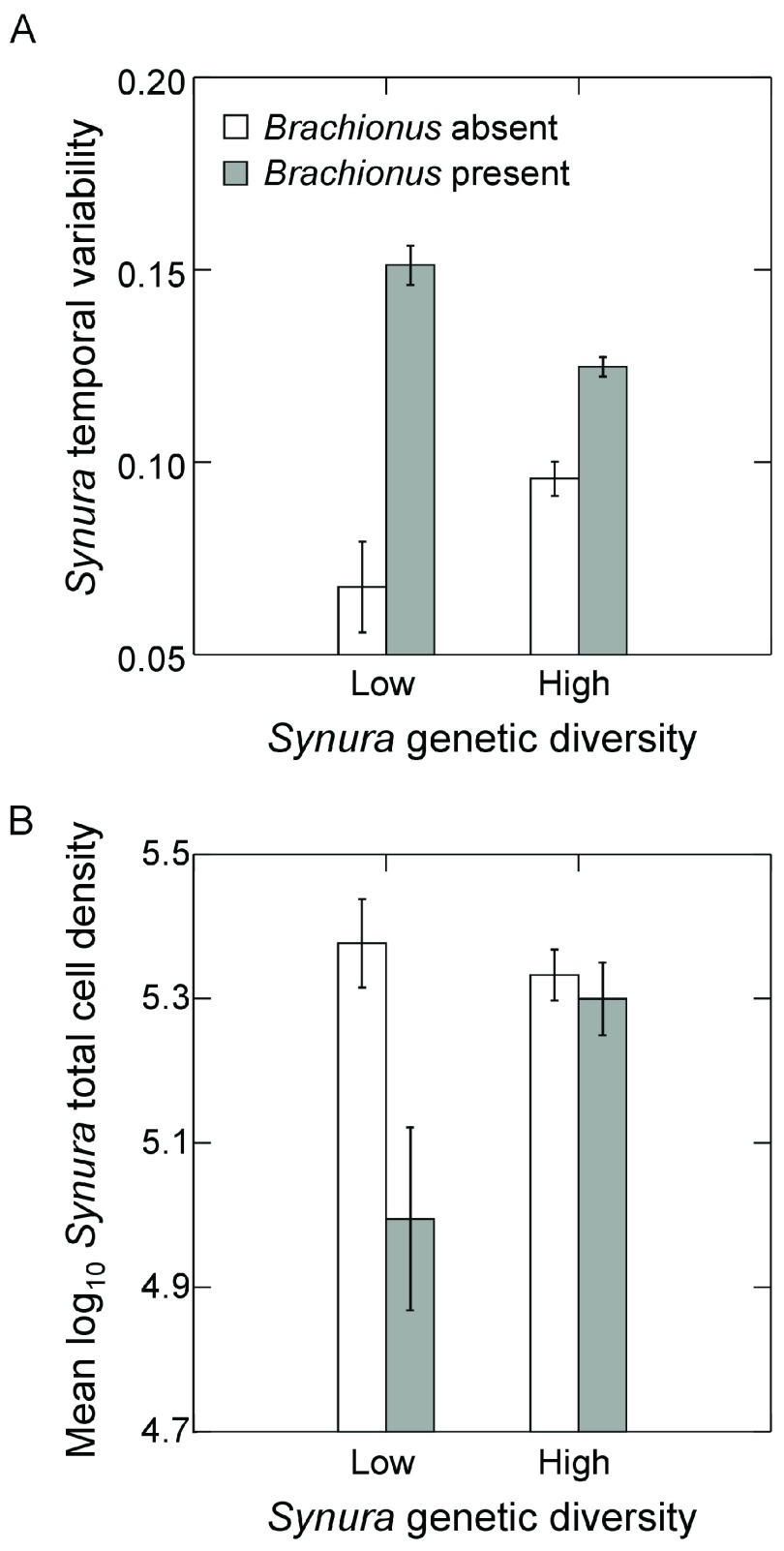
Effects of initial *Synura* genetic diversity (low versus high) and *Brachionus* presence/absence on ( **A**) detrended temporal variability of *Synura* populations (based on total cell densities), measured as mean residuals from linear regressions of log transformed *Synura* density versus time, and ( **B**) *Synura* total cell densities averaged over the course of the experiment.Both measures ( **A** and **B**) were based on dynamics over days 51 to 167. Shown are means (+/- S.E.). Original units in numbers of individuals per mL.

**Figure 6.**
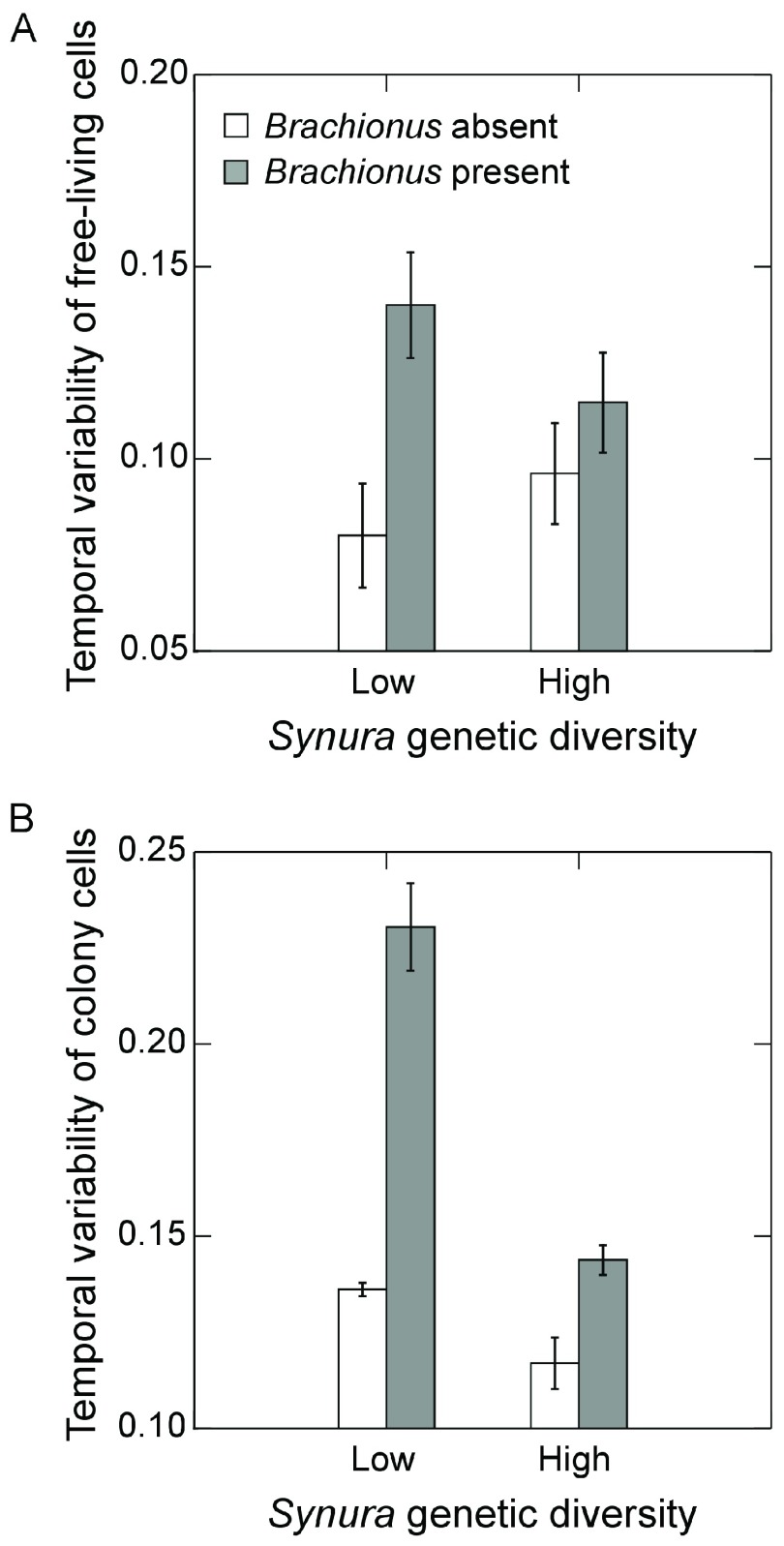
Effects of initial *Synura* genetic diversity (low versus high) and *Brachionus* presence/absence ( **A**) detrended temporal variability of free-living cells of *Synura*, measured as mean residuals from linear regressions of log transformed free cell densities versus time, and ( **B**) detrended temporal variability of colony cells of *Synura*, measured as mean residuals from linear regressions of log transformed colony cell densities versus time. Shown are means (+/- S.E.).

Examining temporal variability of *Synura* total cell densities, a significant interaction between *Brachionus* presence/absence and initial genetic diversity was detected ( Figure 5A; F _1,9_=26.9, p<0.001, ANOVA). Effects of genetic diversity on *Synura* stability were only strongly evident in the presence of *Brachionus*, significantly decreasing temporal variability in the high genetic diversity treatment relative to low genetic diversity ( Figure 5A; p=0.02, max t-test). In contrast, no effects of initial genetic diversity were detected in the absence of *Brachionus* ( Figure 5A; p=0.14, max t-test). We performed additional analyses of temporal variability of free-living cells and colony cell abundances to examine how these components contributed to variability in total cell density. Measures were detrended using linear regressions in the same manner as total cell densities. Temporal variability of free-living cells showed trends that mirrored variability of total cell abundances ( Figure 6A). However, effects of genetic diversity were not strong (main effect: p=0.65, ANOVA; interaction: p=0.10, ANOVA) whereas *Brachionus* significantly increased temporal variability of free-living cells ( Figure 6A; F _1,9_=11.2, p=0.01, ANOVA). Genetic diversity and *Brachionus* presence/absence interactively affected temporal variability of colony cell abundances ( Figure 6B; F _1,9_=39.1, p<0.001, ANOVA). Genetic diversity reduced temporal variability in the presence (p<0.001, max t-test) and absence of *Brachionus* (p=0.053, max t-test), but effects were stronger when *Brachionus* was present ( Figure 6B).

Effects of *Brachionus* presence/absence on total *Synura* cell densities also appeared to be dependent on initial genetic diversity ( Figure 4A versus 4B). Analyzing time-averaged densities, a significant interaction was detected ( Figure 5B; F _1,9_=5.5, p<0.043, ANOVA). While *Brachionus* presence/absence had no effects on mean cell densities in the high genetic diversity treatment ( Figure 5B; p=0.99, max t-test), cell densities were significantly depressed in the presence of *Brachionus* in the low genetic diversity treatment ( Figure 5B; p=0.03, max t-test).

**Figure 7.**
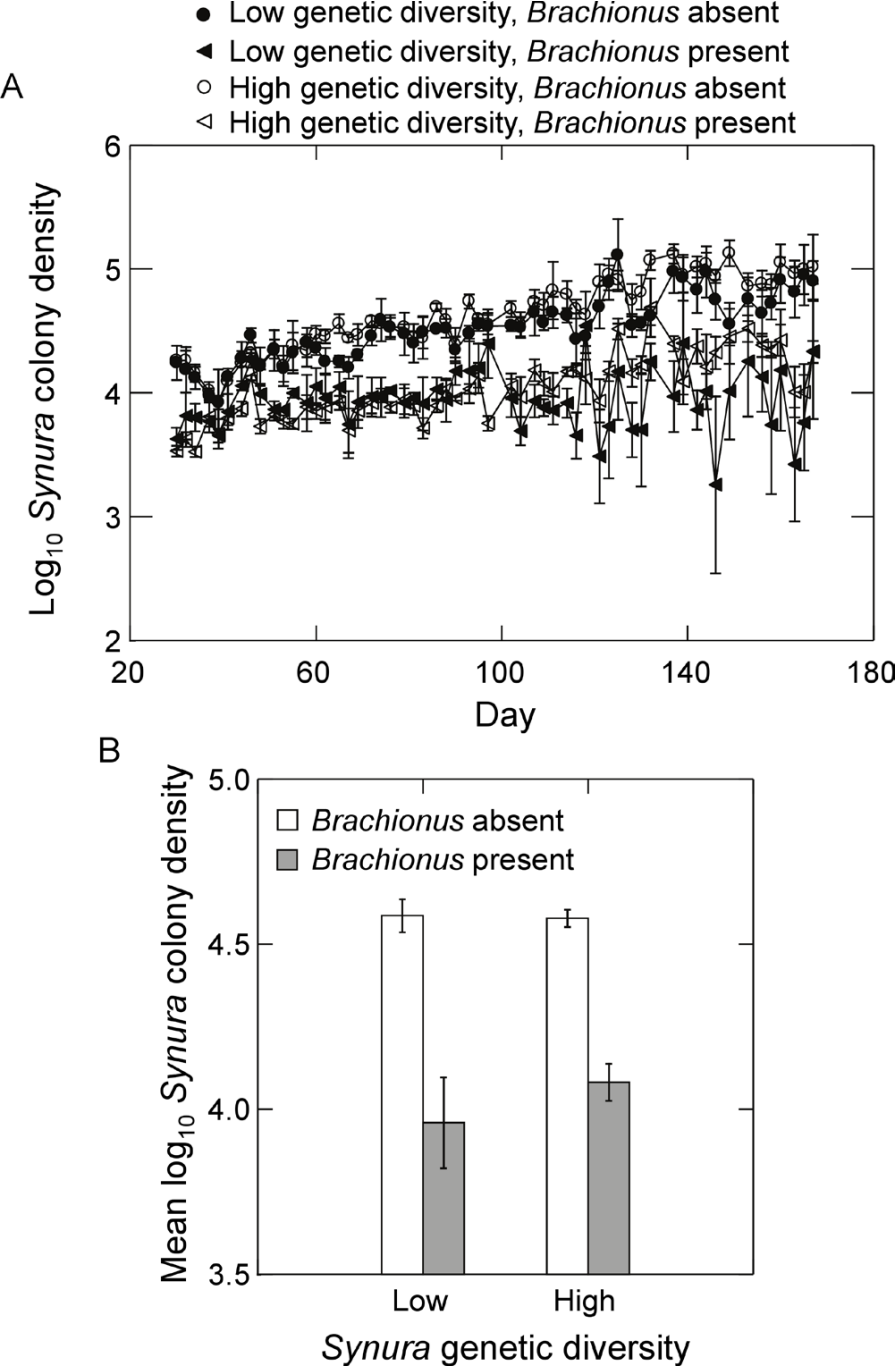
Effects of initial *Synura* genetic diversity (low versus high) and *Brachionus* presence/absence on ( **A**) *Synura* colony densities over time (shown are means across replicates, +/-S.E.), and ( **B**) *Synura* colony densities averaged over days 51 to 167 (means, +/-S.E.).Original units in numbers of colonies per mL.

**Figure 8.**
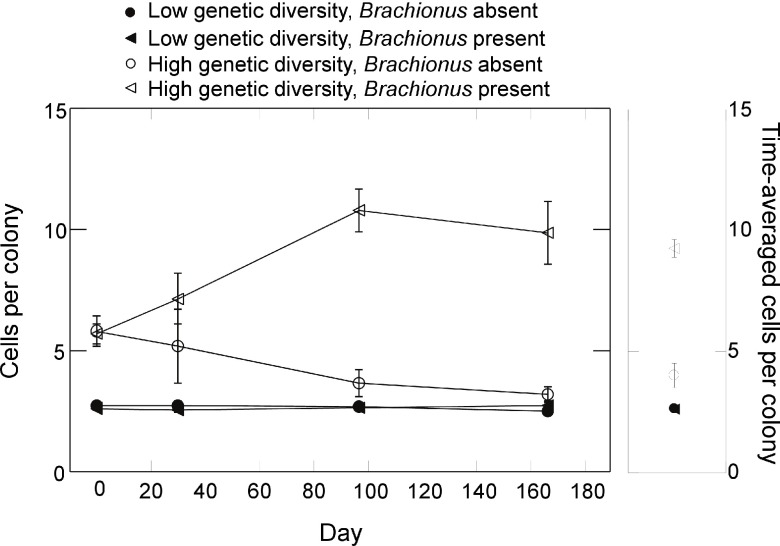
Effects of *Synura* genetic diversity and *Brachionus* presence/absence on the mean number of cells per *Synura* colony. The left panel displays dynamics over time and the right panel time-averaged values (averaged over days 30 to 167). Shown are means (+/- S.E.).

**Figure 9.**
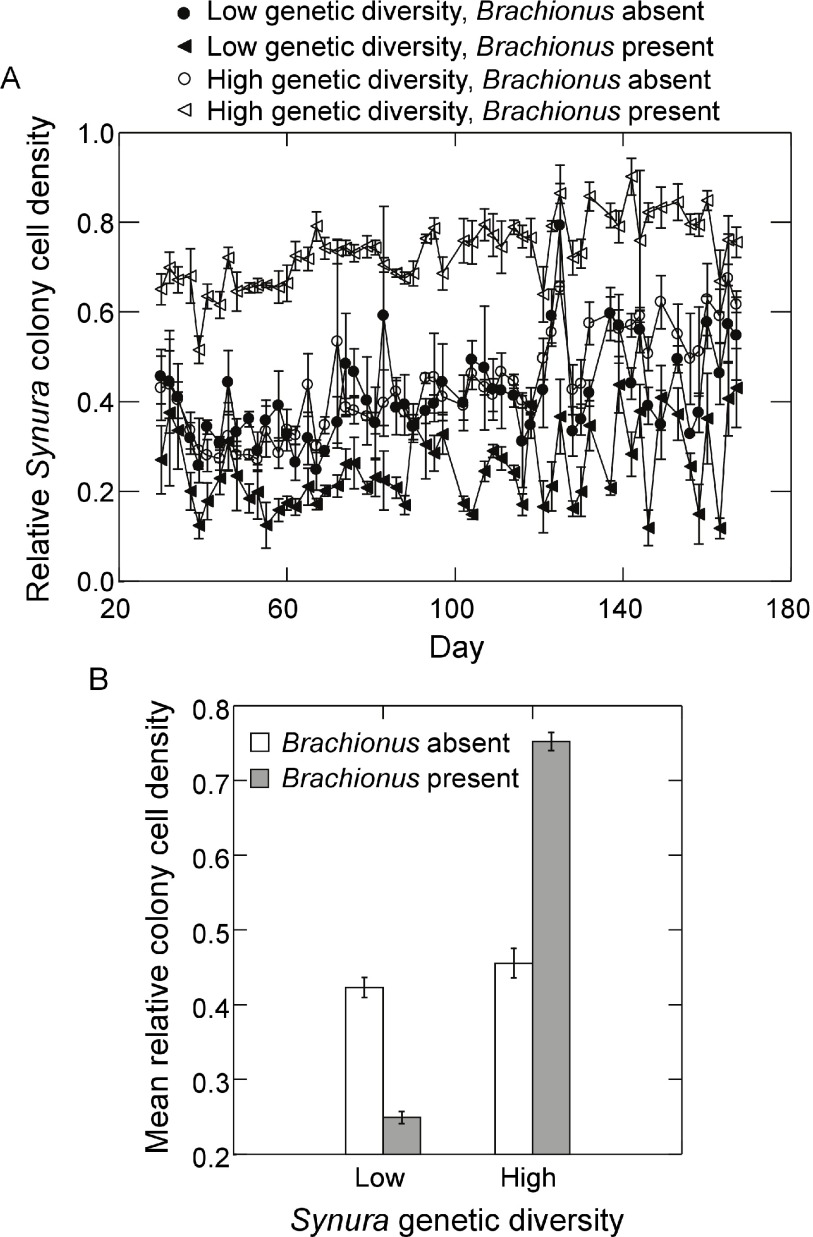
Effects of initial *Synura* genetic diversity (low versus high) and *Brachionus* presence/absence on ( **A**) the relative abundance of *Synura* cells found in colonies over time (shown are means across replicates, +/-S.E.), and ( **B**) the relative abundance of *Synura* cells found in colonies averaged over days 51 to 167 (means, +/-S.E.). Relative colony cell density was calculated by dividing the number of cells found in colonies (per mL) by the total cell density per mL (colony cells plus free-living cells).

Presence of *Brachionus* reduced *Synura* colony density in both genetic diversity treatments ( Figure 7A). When examining time-averaged colony densities, a *Brachionus* effect was present ( Figure 7B; F _1,9_=49.7, p<0.0001, ANOVA) but no main effect of genetic diversity or interaction were detected (p<0.37, ANOVA). While the number of colonies was unaffected by initial genetic diversity, colony size (number of cells per colony) varied greatly between diversity treatments ( Figure 8). When analyzing log transformed time-averages, a significant interaction between genetic diversity and *Brachionus* presence/absence on colony size was detected ( Figure 8; F _1,9_=68.9, p<0.0001, ANOVA). Colony size in the high genetic diversity treatment and in the presence of *Brachionus* was greater compared to all other treatment combinations (all p<0.001, max t-test). Colony size in the high genetic diversity treatment without *Brachionus* was also significantly greater than both low genetic diversity treatments (p=0.03, max t-test). In the low genetic diversity treatment, no effect of *Brachionus* presence/absence was detected (p=0.99, max t-test). Effects on colony size were also apparent when examining the relative number of cells in colonies, which increased greatly in the presence of *Brachionus* and high genetic diversity ( Figure 9A). Analyzing time-averaged relative colony cell density produced a significant interaction between *Brachionus* presence/absence and initial *Synura* genetic diversity ( Figure 9B; F _1,9_=436.1, p<0.0001, ANOVA). There was no difference between low and high genetic diversity treatments when *Brachionus* was not present (p=0.49, max t-test) but a strong positive effect of *Synura* genetic diversity in the presence of *Brachionus* ( Figure 9B; p<0.001, max t-test).

Brachionus synura timeseries Time series for all Brachionus and Synura response variables for all treatments and replicates.

Brachionus and Synura temporal variability and time-averaged densities Time-averaged densities and measures of temporal variability for Brachionus and Synura for all treatments and replicates.

Synura trait assays Results of Synura trait assays (common garden experiments) examining phenotypic variation among the five genotypes of Synura used in the experiment

Synura colony size responses Time series of mean number of cells per Synura colony for all treatments and replicates

## Discussion

We found clear evidence that the presence of intraspecific trait heterogeneity can positively impact the stability and abundance of prey populations when in the presence of predators. These effects were linked to increases in the degree of aggregation of prey individuals into colonies; diverse prey populations produced larger colonies and a greater relative abundance of cells found in colony form when predators were present. Our results bear many similarities to prior studies that have examined the dynamic consequences of interspecific variation in prey defense against predators. As outlined in our Introduction, several models have shown that the presence of weakly interacting prey can stabilize dynamics by shunting resources away from more edible forms and subsequently reducing the amplitude of predator-edible prey oscillations ^3, 5, 23^ – a prediction that has garnered some empirical support from studies that have manipulated prey species composition ^6, 34, 35^ and clonal composition ^15^. Our work further demonstrates that intraspecific variation can stabilize dynamics when prey persist in different dynamic classes that vary in predator resistance. The mechanisms that can give rise to vulnerable-invulnerable class structures within prey populations are diverse and include refuge use, inter-individual variation in behavioral or physiological states, and spatial variation in predation pressure ^1^. Thus, our general findings may have broader applicability beyond planktonic systems of colony-forming prey.

**Figure 10.**
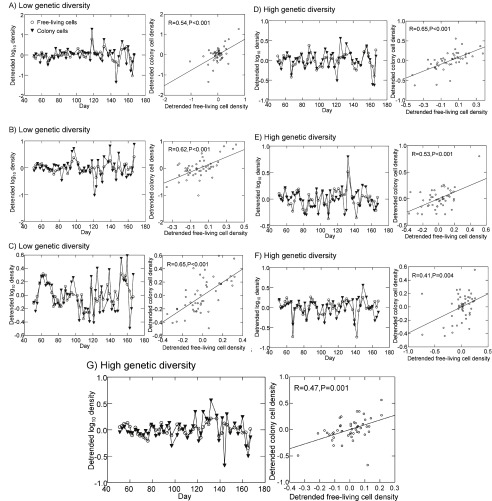
Detrended dynamics of *Synura* free-living cells and colony cell abundances in the presence of *Brachionus* (left panels) for all three replicates of the low genetic diversity treatment ( **A**– **C**) and all four replicates of the high genetic diversity treatment ( **D**– **G**). Shown for each replicate in the right panel is the relationship between free-living cell and colony cell abundances and Pearson correlation statistics (the line is the linear regression fit). Detrended abundances are the residuals from linear regressions of either log free-living cell densities or log colony cell densities versus time.

While stabilization is one potential outcome of enhanced prey diversity, several models have shown that the evolution of prey defense may also destabilize predator-prey dynamics, depending on assumptions of prey and predator traits ^15, 17, 25^. For example, Hairston, Ellner and colleagues have examined the dynamic consequences of evolutionary responses among prey using models and an experimental system composed of *Brachionus calyciflorus* and unicellular green algae as prey ^14– 17^. They have shown both theoretically and experimentally that rapid prey adaption in prey edibility can have significant effects on predator-prey dynamics, though effects are not always stabilizing and dependent on the degree of prey phenotypic variation present and the strength of trade-offs among phenotypes in anti-predator strategies and competitive ability for shared resources. When predator-resistance is effective but costly to defended phenotypes, rapid prey adaptation can induce large predator-prey oscillations and destabilization ^14, 16, 18^. In contrast, when defense against predators is effective but costs in competitive ability are low, prey adaptation can result in cryptic prey cycles in which predators continue to oscillate and total prey abundance is stabilized as edible and predator-resistant phenotypes oscillate out of phase of each other ^15, 18^. The production of cryptic cycles via rapid prey adaptation shows a passing similarity to our results, in which high genetic diversity had no effects on predator stability but led to stabilization of total *Synura* abundance in the presence of *Brachionus*. Because our sampling intervals were uneven, spanning 2 or 3 days, we could not examine covariation between free-living cells and colony cell abundances using traditional cross-correlation analysis. However, visual examination of detrended abundances of free-living *Synura* cells and colony cells over time revealed no strong support for asynchronous oscillations between the two groups ( Figure 10). Dynamics were instead highly synchronous; significant positive correlations were detected for all replicates ( Figure 10).

The absence of cryptic cycles in our experiment is perhaps not surprising as the life history of our prey species is quite different from that assumed in prior models in which different genotypes have fixed traits. As described above, *Synura* can persist in two phenotypic states: susceptible free-living cells or a more predator-resistant colonial stage. Cells can transition between states via cellular aggregation and colony disassembly or subsets of the population can remain within states since both free living cells and colonies can reproduce – the latter through binary fission ^36^. The prey life history in our experimental system is similar to the prey strategy presented in model 1 of ^1^, in which a single prey species transitions between a vulnerable and invulnerable class, both of which may reproduce. Their model, in addition to several variants, shows that class transitions can reduce top-down control of prey and stabilize dynamics by moving predator-prey cycles to point attractors. However ^1^, only focused on a two species system in the absence of prey evolution. Exploration of the eco-evolutionary dynamics of multi-class prey systems may prove fruitful as it seems plausible that many natural prey populations could persist in multiple dynamic classes that vary in their ecological traits.

Our work also produced results that were similar to prior investigations of the consequences of prey heterogeneity on top-down versus bottom-up control of trophic-level production ^2, 4, 7, 10– 12, 37^. These studies have all provided strong empirical evidence that predators can facilitate numerical dominance of defended prey species or phenotypes, reducing predator limitation of total prey production. Similarly, we found that presence of predators selected for dominance by large colony phenotypes with reduced susceptibility to predation pressure. This resulted in a significant increase in mean prey abundance when compared to populations that were initially composed of a single, susceptible phenotype. Hence, prey evolution has the capacity to alter trophic structure and the partitioning of production among trophic levels.

As with any experiment, there are caveats and limitations in our study that deserve mention. First, it is important to note that our contrast between low and high prey genetic diversity was performed with a single genotype (CBS) for the low diversity treatment. This is an approach that has been used in prior experiments in which dynamics in the presence of a highly edible prey phenotype are contrasted with dynamics in which the edible prey has been supplemented with additional phenotypes that vary in their susceptibility to predators ^2, 7, 9, 16^. In the present case, this was done out of necessity since we observed that the CBS *Synura* strain was the only one capable of maintaining *Brachionus* populations at densities that were not prone to rapid extinction. This was likely due to the CBS strain’s combination of high cell density, small colony size and low relative abundance of cells in colonies compared to the other strains ( Figure 1). The only exception to this was strain LB239, which also had small colonies and a low relative abundance of cells in colony form. However, this strain also exhibited the lowest cell densities among the prey genotypes ( Figure 1), which likely contributed to predator extinctions. If using extinction probability as a measure of predator stability, our results bolster our argument that prey genetic diversity enhances stability; in monocultures of *Synura*, *Brachionus* extinction probability was high for four out of five strains but low in the prey polyculture. Another limitation of our study is that we did not measure competitive abilities among our *Synura* strains. A central assumption of many of the abovementioned models is that prey exhibit trade-offs in their ability to resist predation and their competitive ability for shared resources ^3, 11, 13, 14, 23, 26, 38, 39^. Hence, investment in defensive traits - whether behavioral, chemical or morphological – comes at a cost to the organism in terms of its ability to acquire or convert limiting resources to growth and reproduction. While we did not measure competition between our *Synura* strains, it is conceivable that colony aggregation and increasing colony size could incur a fitness cost on individual cells by reducing exposed cell surface area and impairing the ability of individuals to acquire nutrient resources. Assuming a single limiting resource, models predict that phenotypes with high competitive ability should exclude less competitive, defended phenotypes at equilibrium and in the absence of predators. Results from our experiment were consistent with this prediction. In the absence of *Brachionus*, mean colony size and the relative abundance of *Synura* cells in colonies fell to low levels that were similar in magnitude to the low diversity treatment. This is indicative of a shift in dominance to phenotypes with lower investment in defense.

Cellular aggregation is viewed as an evolutionary stepping stone between unicellular and multicellular life forms ^28, 30, 40^. A hurdle in achieving this initial transition is the fitness costs associated with colony formation such as reductions in competitive ability, reproductive rates or buoyancy. Our work supports the general idea that colony formation and large colony size provide a selective advantage in the presence of predators and a disadvantage when predators are absent and resource competition is strong. This finding complements previous studies that have shown similar effects of predators on colony formation in unicellular algae ^17, 41^ and supports the general hypothesis that a driver in the early evolution of multicellularity was the emergence of heterotrophic life forms and phagotrophy – a transition point where the disadvantages of cellular aggregation began to be outweighed by the advantages of increasing size and predator escape ^29^. Our study also adds to a growing body of experiments demonstrating the effects of rapid evolutionary change on the dynamics of consumer-resource interactions ^2, 16, 17, 27, 42^ and the strength of top-down control of prey abundance ^2, 37^. How such effects translate to natural communities remains largely unresolved; the majority of prior experiments, including ours, have utilized highly simplified laboratory settings with a minimal number of interacting species. Moreover, the consequences of coevolutionary responses between predator and prey populations on ecological dynamics have not been deeply explored. Natural prey populations are of course exposed to multiple species of predators and embedded in prey communities with a large degree of interspecific variation in competitive ability and defense against predators. Understanding the dynamic consequences of evolution and coevolution within the context of complex communities remains a daunting challenge in ecology but an exciting avenue for future exploration.
