# Supplementary material for: The stabilizing effects of genetic diversity on predator-prey dynamics
Source: F1000Res. 2013 Feb 12;2:43. [Version 1] doi: 10.12688/f1000research.2-43.v1 (PMC4193400; doi:10.12688/f1000research.2-43.v1)
Supplement: Brachionus synura timeseries — Time series for all Brachionus and Synura response variables for all treatments and replicates. [file f1000research-2-354-s0000.tgz › brachionus_synura_means_tempvariation_column_headers_codes.docx]

**Explanation of column headers and codes:**

BRACH_TRT - predator treatment; “present” corresponds to the presence of the predator (*Brachionus*); “absent” corresponds to the absence of the predator

DIV_TRT - prey diversity treatment; “Monoculture” corresponds to treatments with only the CBS strain of *Synura* present; “Polyculture” corresponds to treatments with all five strains of *Synura* initially present.

REP - numbered replicate identifier

LOGBRACHDENS - log_10_ transformed *Brachionus* density (individuals per mL) averaged over time

BRACHTEMPVAR -*Brachionus* temporal variability measured as the average of the absolute values of the residuals from linear regressions of log_10_ transformed *Brachionus* density versus time

LOGTOTSYNDENS - log_10_ transformed total cell density of *Synura* (per mL) averaged over time

LOGSYNCOLDENS - log_10_ transformed density of *Synura* colonies (per mL) averaged over time

RELSYNCOLCELLDENS - relative abundance (proportion) of *Synura* cells found in colonies averaged over time

SYNTOTTEMPVAR - temporal variability of total *Synura* density measured as the average of the absolute values of the residuals from linear regressions of log_10_ transformed *Synura* total cell density versus time

SYNFREECELLTEMPVAR- temporal variability of total *Synura* density measured as the average of the absolute values of the residuals from linear regressions of log_10_ transformed *Synura* total free cell density versus time

SYNCOLCELLTEMPVAR- temporal variability of total *Synura* density measured as the average of the absolute values of the residuals from linear regressions of log_10_ transformed density *Synura* cells found in colonies versus time
