# Supplementary material for: The stabilizing effects of genetic diversity on predator-prey dynamics
Source: F1000Res. 2013 Feb 12;2:43. [Version 1] doi: 10.12688/f1000research.2-43.v1 (PMC4193400; doi:10.12688/f1000research.2-43.v1)
Supplement: Brachionus and Synura temporal variability and time-averaged densities — Time-averaged densities and measures of temporal variability for Brachionus and Synura for all treatments and replicates. [file f1000research-2-354-s0001.tgz › brachionus_synura_timeseries_column_headers_codes.docx]

**Explanation of column headers and codes:**

day - day of the experiment

div_trt - prey diversity treatment; “Monoculture” corresponds to treatments with only the CBS strain of *Synura* present; “Polyculture” corresponds to treatments with all five strains of *Synura* initially present.

brach_trt - predator treatment; “present” corresponds to the presence of the predator (*Brachionus*); “absent” corresponds to the absence of the predator

rep - numbered replicate identifier

logbrachdens - log_10_ transformed *Brachionus* density (individuals per mL)

synrelcolcelldens - relative abundance (proportion) of *Synura* cells found in colonies

lsyntotcelldens - log_10_ transformed total cell density of *Synura* (per mL)

lsyncoldens - log_10_ transformed density of *Synura* colonies (per mL)

lsyncolcelldens - log_10_ transformed density of *Synura* cells found in colonies (per mL)

brachresid - absolute values of the residuals from linear regressions of log_10_ transformed *Brachionus* density versus time

syntotcellresid - absolute values of the residuals from linear regressions of log_10_ transformed *Synura* total cell density versus time

synfreecellresid - absolute values of the residuals from linear regressions of log_10_ transformed *Synura* total free cell density versus time

syncolcellresid - absolute values of the residuals from linear regressions of log_10_ transformed density *Synura* cells found in colonies versus time
