# Supplementary material for: The stabilizing effects of genetic diversity on predator-prey dynamics
Source: F1000Res. 2013 Feb 12;2:43. [Version 1] doi: 10.12688/f1000research.2-43.v1 (PMC4193400; doi:10.12688/f1000research.2-43.v1)
Supplement: Synura trait assays — Results of Synura trait assays (common garden experiments) examining phenotypic variation among the five genotypes of Synura used in the experiment [file f1000research-2-354-s0002.tgz › Synura_assays_timeseries_column_headers_codes.docx]

**Explanation of column headers and codes:**

day - day of the assay

strain - identity of the *Synura* strain ( LB239, LB2403, LB2405, LB2406,CBS)

rep - numbered replicate identifier

logfreecelldens - log_10_ transformed *Synura* total free cell density (per mL)

logcoldens - log_10_ transformed density of *Synura* colonies (per mL)
