# Supplementary material for: The stabilizing effects of genetic diversity on predator-prey dynamics
Source: F1000Res. 2013 Feb 12;2:43. [Version 1] doi: 10.12688/f1000research.2-43.v1 (PMC4193400; doi:10.12688/f1000research.2-43.v1)
Supplement: Synura colony size responses — Time series of mean number of cells per Synura colony for all treatments and replicates [file f1000research-2-354-s0003.tgz › synura_colony_size_overtime_column_headers_codes.docx]

**Explanation of column headers and codes:**

day - day of the assay

div_trt - prey diversity treatment; “Monoculture” corresponds to treatments with only the CBS strain of *Synura* present; “Polyculture” corresponds to treatments with all five strains of *Synura* initially present.

brach_trt - predator treatment; “present” corresponds to the presence of the predator (*Brachionus*); “absent” corresponds to the absence of the predator

rep - numbered replicate identifier

cellspercolony - mean number of cells per *Synura* colony
