# Supplementary material for: The stabilizing effects of genetic diversity on predator-prey dynamics
Source: F1000Res. 2013 Feb 12;2:43. [Version 1] doi: 10.12688/f1000research.2-43.v1 (PMC4193400; doi:10.12688/f1000research.2-43.v1)
Supplement: Peer review history

# Peer review: Referee response for version 1

**DOI:** 10.5256/f1000research.354.r4251

**Published:** 2014-04-09

**Authors:** Jordi Moya-Laraño

**Affiliations:** 1 Functional and Evolutionary Ecology, Estación Experimental de Zonas Áridas, Almería, Spain

This article shows how increasing genetic variation in prey can stabilize the dynamics, especially since predator can barely establish in communities without variability in prey. This last finding is by far the strongest and I think that it adds substantially to a current important topic. A second relevant finding is that, despite having only a single strain with low variability, the high genetic variability replicates had also more stable dynamics than the one single strain.

The finding that colony formation was linked to predator presence and that there was a trend in which colony formation increased through time also provides a hint on how multicellularity evolved.

However, unfortunately I do not think that this article provides convincing evidence of an evolutionary response of defense through colony formation as the authors claim. First, in their preliminary assays the authors did not combine the different strains to see how they form colonies when different strains are present in the same habitat. We do not know if they are able to combine and to cooperate with other strains (at least with the information provided) and if there are emergent patterns of colony formation when more than one strain is combined.

The findings that defense (aggregative behaviour) increase when predators are present could be just the result of complex patterns of (plastic) cooperation among strains which change through time, for instance as a response to persistent predation risk. Assays at the end of the experiment to test if after removing predators, colonies were still maintained at substantially high rates (e.g. as in strain LB2405) would have been a convincing evidence for adaptive evolution ocurring during the dynamics.

I have read this submission. I believe that I have an appropriate level of expertise to confirm that it is of an acceptable scientific standard.

# Peer review: Referee response for version 1

**DOI:** 10.5256/f1000research.354.r2471

**Published:** 2013-11-18

**Authors:** Gregory Crutsinger

**Affiliations:** 1 Department of Zoology, University of British Columbia, Vancouver, BC, Canada

The authors of this study performed a laboratory mesocosm experiment that sought to address the role of intraspecific variation in predator-prey dynamics. While the underlying questions presented an interesting and relevant avenue of research, four of the five clonal lines went extinct in their single-genotype treatment.  As a result, their contrasts between low and high prey genetic diversity were performed with a single genotype (CBS strain). Consequently, it is unclear to me whether conclusions could be drawn about the consequences of genetic diversity *per se*, as opposed to the identity effects of this individual genotype. I agree with the authors that it is a major limitation in interpreting this experiment.

I have read this submission. I believe that I have an appropriate level of expertise to confirm that it is of an acceptable scientific standard, however I have significant reservations, as outlined above.

# Peer review: Referee response for version 1

**DOI:** 10.5256/f1000research.354.r767

**Published:** 2013-02-13

**Authors:** Eva Kisdi

**Affiliations:** 1 Department of Mathematics and Statistics, University of Helsinki, Helsinki, Finland

This paper demonstrates that prey heterogeneity stabilizes prey population dynamics in an experimental predator-prey system. It should, however, be noted that if this happens via the better-defended prey shunting away resources from the edible prey, then the density of the predator should be lower in the high-prey-diversity treatment. But, according to Fig 3B, this is not the case.

I have read this submission. I believe that I have an appropriate level of expertise to confirm that it is of an acceptable scientific standard.
